# DDX10 RNA Helicase: Structure, Function, and Oncogenic Roles Across Solid and Hematologic Tumors

**DOI:** 10.3390/genes17020138

**Published:** 2026-01-27

**Authors:** Giorgia Isinelli, Genny Scacci, Arianna Capocchia, Carla Emiliani, Cristina Mecucci, Roberta La Starza, Danika Di Giacomo

**Affiliations:** 1 Department of Chemistry, Biology and Biotechnology, University of Perugia, 06100 Perugia, Italy; giorgia.isinelli@unipg.it (G.I.); genny.scacci@dottorandi.unipg.it (G.S.); arianna.capocchia@studenti.unipg.it (A.C.); carla.emiliani@unipg.it (C.E.); 2 Hematology and Bone Marrow Transplantation Unit, Department of Medicine and Surgery, CREO, Azienda Ospedaliera di Perugia, University of Perugia, 06100 Perugia, Italy; cristina.mecucci@unipg.it (C.M.); roberta.lastarza@unipg.it (R.L.S.)

**Keywords:** DDX10, DEAD-box RNA helicase, ribosome biogenesis, NUP98::DDX10 fusion, acute myeloid leukemia (AML), solid tumors, liquid–liquid phase separation (LLPS), epigenetic regulation, oncogenic transformation, tumor biomarker

## Abstract

DEAD-box (DDX) RNA helicases are essential regulators of RNA metabolism and gene expression. Among them, DDX10 remains poorly characterized despite growing evidence supporting its involvement in human diseases. This review provides a comprehensive analysis of DDX10, from its structural and functional features to its emerging roles in solid tumors and hematologic malignancies. We discuss how DDX10, through its conserved domains, contributes to pre-rRNA processing, ribosome biogenesis, and cell proliferation, and explore potential links between DDX10 and processes such as liquid–liquid phase separation (LLPS) and epigenetic regulation, which may underlie its roles in cancer cell plasticity and stress response. We argue that the dysregulation of these fundamental cellular processes positions DDX10 as a focal point where aberrant RNA metabolism and altered molecular condensates converge to disrupt transcriptional homeostasis and drive oncogenic transformation. Aberrant DDX10 expression is a recurrent feature across multiple cancers, where it promotes tumor progression, therapy resistance, and poor prognosis. Moreover, DDX10 participates in oncogenic fusion events, most notably the NUP98::DDX10 fusion identified in a subset of acute myeloid leukemias, which drives leukemogenesis by disrupting transcriptional regulation and cellular differentiation. Given its tumor-associated expression and diverse biological functions, DDX10 is increasingly recognized as a potential diagnostic biomarker and a promising target for therapeutic strategies. By consolidating current knowledge under this unifying framework, this review highlights the multifaceted roles of DDX10 in cancer biology, advocating further research into its molecular functions and translational potential.

## 1. Introduction

DDX helicases constitute a family of Adenosine Triphosphate Protein (ATP)-dependent RNA helicases, classified under the helicase superfamily 2 (SF2), which is the largest family of nucleic acid helicases [1]. DEAD-box RNA helicases possess a highly conserved core region characterized by twelve signature motifs, including the conserved Asp-Glu-Ala-Asp (DEAD) motif. These elements are crucial for their ATPase activity and RNA-unwinding functions, which define their role as RNA helicases [2]. They perform various, often critical, functions related to RNA processing, such as splicing, ribosome biogenesis, translation initiation, transport, and turnover. They also play a role in stress by contributing to the formation of stress granules, non-membranous organelles that form when translation is impaired [3], and in innate immunity by recognizing structural features characteristic of viral RNAs, leading to the induction of interferon and interferon-stimulated genes [4,5]. Many DDX proteins are multifunctional, participating in a wide range of cellular processes beyond RNA unwinding. These additional roles are largely attributed to their variable, less conserved N- and C-terminal domains, which mediate specific interactions with partner proteins [6]. DEAD-box proteins participate in critical processes related to cellular proliferation and malignant transformation. Consequently, dysregulation in their expression or function can disrupt normal cellular homeostasis and contribute to cancer development and progression, as demonstrated by the aberrant expression profiles of several DEAD-box helicases in high-risk malignancies, including Philadelphia-like B-lineage acute lymphoblastic leukemia [7], AML and myelodysplastic syndrome [8], prostate cancer [9], gastric cancer [10], breast cancer [11] and others [12,13,14,15]. Depending on the context, DEAD-box helicases can function either as oncogenes or tumor suppressors across different types of cancer [16,17,18]. Among these, DDX10 is emerging as a particularly multifaceted player. In this review, we argue that DDX10 serves as a critical nexus where RNA metabolism, liquid–liquid phase separation (LLPS), and transcriptional regulation converge to drive oncogenic transformation. This review offers a comprehensive overview of the RNA helicase DDX10 beginning with its physiological roles and molecular characteristics and then examining its involvement in solid and hematologic malignancies, focusing on its fusion with the *NUP98* gene. It concludes by addressing the potential of DDX10 as a diagnostic biomarker and as a promising target for selective anticancer therapies, highlighting how targeting this molecular hub could disrupt the coordinated processes that sustain cancer cell plasticity and progression.

## 2. Structural Basis of DDX10 Function Within the DEAD-Box Helicase Family

The functionality of DEAD-box helicases relies on three essential and interdependent processes: ATP binding, ATP hydrolysis, and selective recognition of RNA substrates, which together drive the unwinding of RNA secondary structure [19]. DDX proteins share a highly conserved helicase core, while variable auxiliary domains account for the differences among family members and are crucial in defining the specific functions of each enzyme [19].

**Figure 1:**
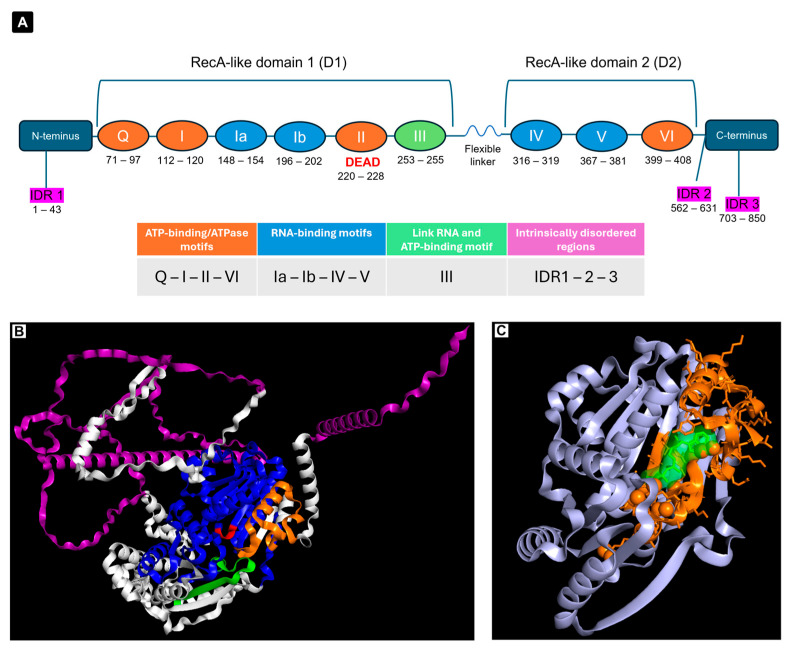
**Structural representation of DEAD-box helicases.** Schematic illustration of the conserved motifs and domain organization. The domain numbering and amino acid coordinates specifically refer to DDX10, the primary subject of this review. (**A**) Schematic representation of the two RecA-like domains (D1 and D2) of the DDX10 protein. Orange indicates the ATP-binding/ATPase motifs (Q, I, II and VI), blue marks the RNA-binding motifs (Ia, Ib, IV and V), green denotes the motif III connecting RNA- and ATP-binding domains, and fuchsia shows the IDRs. The conserved DEAD sequence is highlighted in red. (**B**) 3D structural model of DDX10 generated with PyMOL 3.1.6.1 [21], adopting the same color scheme as in panel A. The IDRs are shown with low confidence due to limited structural data, consistent with predictions from AlphaFold. (**C**) Close-up view of the ATP-binding pocket. The ATP-binding pocket is shown in orange, and green represents an ATP molecule. The terminal spheres on the sticks represent amino acid residues directly involved in ATP interaction. Non-relevant motifs were removed—for clarity in (**C**). DDX10’s crystallographic structure (AF-Q80Y44-F1-v4) was retrieved from AlphaFold (AF-Q80Y44-F1-v4, https://alphafold.ebi.ac.uk/, accessed on 25 June 2025) and loaded on PyMOL to highlight its domains and binding site.

The helicase core consists of two recombinase A (RecA)-like domains connected by a flexible linker, facilitating changes in their reciprocal orientation as shown in Figure 1A. The N-terminal domain (domain 1, D1) encompasses motifs Q, I (also referred to as “A”), Ia, Ib, Ic, II (also referred to as “B”), and III; and the C-terminal domain (domain 2, D2) comprises motifs IV, IVa, V, Va, and VI [20] (Figure 1A,B). Motifs from both domains interact with ATP and RNA. Specifically, ATP interacts with motifs Q, I, II, and VI (Figure 1C) while RNA interacts with motifs Ia, Ib, Ic, IV, IVa, and V [20].

The interplay between the two RecA-like domains is considered crucial for coordinating ATP binding and hydrolysis, which in turn influences changes in RNA binding affinity and the final unwinding of the target RNA [19].

**Figure 2:**
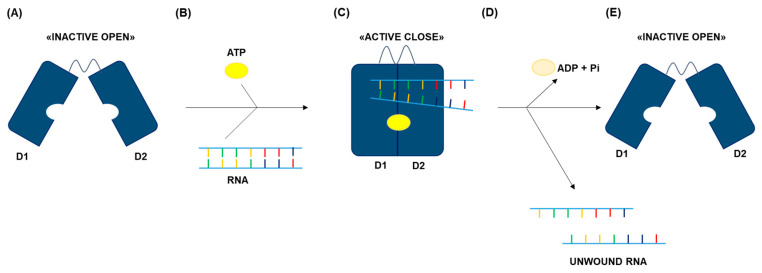
**Schematic representation of the DEAD-box RNA helicases catalytic cycle.** (**A**) In the open state, the two RecA-like domains (D1 and D2) are separated. (**B**) Cooperative binding of RNA and ATP occurs in the open conformation. (**C**) Ligand binding induces domain closure, causing RNA displacement and generating the catalytically active state, enabling ATP hydrolysis. (**D**) ATP hydrolysis triggers RNA, ADP and inorganic phosphate (Pi) release. (**E**) Product release allows reopening of the enzyme and resets the catalytic cycle.

This coordination is driven by a series of conformational changes. Specifically, in the absence of ATP and RNA, the helicase cores are maintained in an “inactive open” conformation, allowing them to move freely without forming interdomain interactions [22]. ATP can bind the open inactive form [22], a process coordinated by the Q motif, which ensures adenine specificity, and Motif I, which interacts directly with the β- and γ-phosphates of ATP [20,23,24,25]. Concurrently, motifs Ia, Ib and IV in both RecA-like domains, engage the RNA substrate via a highly positively charged surface cleft, interacting with the negatively charged sugar-phosphate backbone in a sequence-independent manner [20,24,26]. Importantly, RNA binding occurs in an arch-like shape, which is incompatible with RNA duplex and thus causes the eviction of one RNA strand [27,28,29,30,31,32]. These RNA interactions, together with ATP binding, stimulate a conformational transition of the helicase cores to the “active closed” conformation [33]. Although the precise structural communication between the ATP and RNA binding sites remains incompletely understood, motif III senses and stabilizes the conformational closure of the two RecA-like domains, coupling ATP binding to structural rearrangements [34,35,36]. At this stage, ATP hydrolysis occurs, primarily mediated by motif II (DEAD) [2,20], while motifs V and VI coordinate interactions between the ATP γ -phosphate and the RNA backbone, transmitting the energy derived from ATP hydrolysis to locally destabilize the RNA [20,37]. Motif VI additionally helps sensing the ATP state, facilitating the reopening of the helicase cleft [20,24]. This conformational change reduces RNA affinity, allowing the helicase to release the RNA, but also the ADP + Pi completing a single catalytic cycle [33,37,38,39] (Figure 2).

Auxiliary domains flank the N- and C-termini of the helicase core and often surpass the core itself in size. These regions often adopt well-defined structural folds and perform specific functions, such as nuclease activity, RNA binding, and recruitment of protein complexes [22,27].

Among DEAD-box helicases, DDX10 is an 875 amino acid protein with an approximate molecular weight of 100.9 kDa [40], encoded by a gene located on chromosome 11q22.3 [40]. The protein structure was described in detail by Schütz et al. [41]. DDX10 contains several conserved motifs characteristic of DEAD-box helicases: motif Q (FSDFPLSKKTLKGLQEAQYRLVTEIQK), motif I (AKTGSGKT), motif II (DEAD), and motif VI (YIHRAGRTAR), which are essential for ATPase activity and ATP hydrolysis, processes that drive RNA unwinding. In contrast, motifs Ia (PTRELA), Ib (TPGRLL), IV (SIVF), and V (VLFATDIAARGLDF) are primarily involved in RNA binding, while motif III (SAT) plays a key role in coupling ATP hydrolysis to RNA unwinding [40,41,42]. In addition, DDX10 possesses three intrinsically disordered regions (IDRs) [43] (Figure 1A,B), which provide the protein with structural flexibility and may facilitate LLPS, a crucial mechanism underlying the formation of membraneless organelles through dynamic macromolecular interactions [44].

## 3. The Physiological Functions of DDX10

DDX10 localizes to the nucleolar Dense Fibrillar Component (DFC) and Granular Component (GC) of the nucleolus [43]. Its major role in ribosome assembly largely accounts for its widespread expression [40]. In mouse embryonic stem cells (mESCs), where ribosome biogenesis is essential to preserve stem cell identity [45,46,47], DDX10 is highly expressed and downregulated upon differentiation [43]. Rapid DDX10 degradation using a CRISPR-Cas9 degron system results in reduced proliferation, G1 cell cycle arrest, and apoptosis on mESCs [43].

Mechanistically, DDX10 binds the 45S rRNA subunit [43]. Loss of DDX10, or its yeast homolog Dbp4, impairs snoRNA release from pre-ribosomes (U3 in mESCs [43] and U14 in yeast [48]), thereby blocking 18S ribosomal RNA maturation and compromising ribosome biogenesis [43].

Recent findings also support a role for DDX10 in promoting the initial stages of ribosome biogenesis through LLPS [43]. This process, which drives the formation of membraneless organelles through dynamic macromolecular interactions, is mediated by three IDRs in the DDX10 protein (Figure 1A,B), enabling its condensation into liquid-like droplets that scaffold early steps of ribosome assembly [43]. The ability of DDX10 to undergo LLPS is also exploited in certain pathological contexts, as will be further discussed in the review.

Beyond its role in ribosome biogenesis, DDX10 contributes to the host antiviral response. Its activity occurs in infections with porcine reproductive and respiratory syndrome virus (PRRSV) and porcine circovirus type 3 (PCV3) [49,50]. Ectopic expression of DDX10 enhances the production of type I interferon, which activates the expression of interferon-stimulated genes (ISGs) to suppress viral replication [49,50], whereas DDX10 depletion enhances viral proliferation [50]. Further studies revealed that the N-terminal nuclear localization signal (NLS) of the capsid (Cap) protein in PCV3 interacts with the helicase domain of DDX10. This interaction is crucial for amplifying the DDX10-mediated antiviral response [50].

The role of DDX10, however, appears to be context-dependent, as it may promote rather than suppress viral replication during human immunodeficiency virus 1 (HIV-1) infection. While the exact mechanism remains unclear, an increase in the expression of DDX10, alongside other DDX helicases, has been observed; this suggests that HIV-1 exploits the role of DDX10 in ribosome biogenesis, thereby boosting the protein synthesis machinery essential for efficient viral replication [51,52,53].

## 4. DDX10: A Recurrent Pan-Tumoral Oncogene

The emerging role of DDX10 in cancer suggests that it acts as a central molecular nexus, where distinct biochemical activities converge to drive malignancy. Its oncogenic potential is not limited to a single pathway but is built upon three functional pillars: the modulation of proliferative signaling, the spatial organization of the cellular environment through phase separation, and the maintenance of translational capacity via ribosome biogenesis. In the following sections, we discuss how the aberrant upregulation and structural alterations of DDX10 exploit these mechanisms to promote tumor progression and therapy resistance.

### 4.1. Aberrant DDX10 Upregulation as a Common Feature in Different Human Cancers

DDX10 is consistently overexpressed across multiple malignancies, including colorectal cancer (CRC) [54,55,56], osteosarcoma [57,58], lung adenocarcinoma (LUAD) [59,60], pancreatic ductal adenocarcinoma (PDAC) [61], diffuse large B-cell lymphoma (DLBCL) [62], and bladder cancer [63]. In these tumors, elevated DDX10 expression correlates with advanced tumor stage, increased metastatic potential, and poor overall survival, establishing DDX10 as a prognostic biomarker with both diagnostic and biological significance. Several mechanisms have been identified through which DDX10 overexpression contributes to tumorigenesis, highlighting its multifaceted role in cancer progression. The diverse oncogenic roles of DDX10 across these malignancies suggest that it does not act through a single pathway, but rather functions as a molecular hub that integrates multiple survival signals.

In CRC cell lines, DDX10 promotes progression through its interaction with the ribosomal protein RPL35, thereby regulating its splicing and maintaining E2F-dependent transcriptional activity, which is critical for cell cycle progression [56]. Accordingly, silencing DDX10 in this context impairs proliferation, induces apoptosis, and reduces metastatic potential. In addition, DDX10 promotes tumorigenesis by negatively regulating the autophagy-related gene ATG10, thereby suppressing autophagy and further enhancing cancer cell survival and progression [54,55]. Consistent with these data, DDX10 knockdown leads to increased ATG10 expression, reactivation of autophagy, and a marked reduction in cancer stemness and tumorigenic potential, which can be restored by blocking autophagy, either pharmacologically (e.g., with 3-MA) or genetically (e.g., via ATG10 siRNA) [54,55,64].

Specific tumorigenic mechanisms have been found in osteosarcoma [57,58], glioma [65], bladder cancer [63], and PDAC [61], where DDX10 can alter proliferative signaling pathways to sustain cancer growth. Specifically, in osteosarcoma cells (MG63), targeted silencing of DDX10 inhibits the mitogenic cascade mediated by the activation of the MAPK pathway [66,67,68], whereas DDX10 could contribute to glioma tumorigenesis through activation of Akt/NF-κB and Wnt/β-catenin proliferative pathways [69,70,71,72]. In DLBCL, DDX10 is overexpressed together with FBL, physically interacts with it, and promotes malignant growth. Silencing either gene reduces proliferation and invasion and downregulates β-catenin, cyclin D1, and c-Myc, indicating an FBL-dependent mechanism that drives DDX10-mediated tumor progression [62]. These signaling interactions illustrate how DDX10 influences the transcriptional landscape of the cell, representing the first pillar of the proposed framework by linking RNA processing to the activation of major proliferative pathways such as Wnt/β-catenin and MYC.

In bladder cancer, DDX10 is associated with the upregulation of mTORC1 [73], MYC targets [64,73], and epithelial–mesenchymal transition (EMT) signatures [63]. In PDAC, it activates the PI3K-AKT and HIF-1 signaling pathways and drives glycolytic reprogramming. Additionally, DDX10 promotes proliferation in pancreatic cancer through the regulation of ribonucleotide reductase M2 [74], highlighting its involvement in adapting cellular metabolism to the harsh conditions of the tumor microenvironment [61].

In melanoma, DDX10 is part of a gene signature associated with type I interferon (IFN) resistance [75]. Although its expression is elevated in IFN-resistant melanoma cells, the mechanistic role of DDX10 remains unclear and may reflect a broader transcriptional program of cellular adaptation rather than a direct effector of IFN resistance. Intriguingly, patients with higher DDX10 levels display improved responses to immune checkpoint inhibitors (ICIs) [76,77] indicating that DDX10 expression may mark tumor cells that are simultaneously resistant to IFN-induced growth inhibition yet more responsive to immunotherapy. These observations suggest that IFN resistance and ICI sensitivity can co-occur, likely reflecting context-specific cellular states rather than antagonistic functions. Overall, this highlights the need for careful interpretation of DDX10 expression: while it correlates with IFN resistance in vitro, its predictive association with ICI response underscores a more complex, context-dependent role, potentially related to RNA processing or transcriptional adaptability, rather than a simple oncogenic or tumor-suppressive effect.

### 4.2. DDX10-Driven Phase Separation and Ribosome Biogenesis as Oncogenic Drivers

In oral squamous cell carcinoma (OSCC) [78], DDX10 forms dynamic nuclear condensates via LLPS, where it interacts with the small GTPase Rab27b, thereby facilitating the compartmentalization of exosomal cargo loading and trafficking [78,79,80]. Disruption of LLPS using 1,6-hexanediol leads to the dissolution of DDX10–Rab27b condensates, resulting in reduced exosome release and intracellular accumulation of immunosuppressive molecules, such as PD-L1 [81,82]. These findings demonstrate that DDX10-driven phase separation is mechanistically essential for immune evasion in OSCC [83]. This process represents the second pillar of the DDX10 nexus, where the formation of nuclear condensates facilitates the spatial organization of oncogenic factors, such as Rab27b and PD-L1, ultimately promoting immune evasion. Consequently, these observations highlight LLPS as a therapeutically targetable vulnerability in tumors with elevated DDX10 activity. In specific contexts, DDX10 oncogenic potential is linked to its essential role in RNA metabolism, particularly in ribosome biogenesis [43]. In LUAD, for instance, DDX10 binds the U3 small nucleolar ribonucleoprotein IMP4, promoting ribosomal RNA maturation [59] and sustaining cell viability [84]. Loss of DDX10 decreases IMP4 levels, leading to growth arrest and apoptosis, while IMP4 overexpression rescues these phenotypes. This interaction in LUAD provides an additional example of our proposed framework: by stabilizing the ribosomal machinery, DDX10 ensures the translational capacity necessary to sustain the metabolic demands of lung cancer cells.

Although DDX10 generally exhibits oncogenic features across multiple cancer types, its role appears highly context-dependent. In ovarian cancer, DDX10 is epigenetically silenced by *miR-155-5p*, and its downregulation promotes cell proliferation through activation of the Akt/NF-κB pathway [69], supporting a tumor-suppressive function in this specific context. This contrast highlights that DDX10 dysregulation can have divergent consequences depending on tissue type, genetic background, and interacting regulatory networks. Rather than reflecting contradictory biology, these observations suggest that the functional impact of DDX10 is modulated by the cellular and epigenetic landscape, emphasizing the need to interpret its expression and activity within each tumor-specific context.

### 4.3. Oncogenic Fusions Involving DDX10 Are Drivers of Malignant Transformation

To date, two oncogenic DDX10 fusion proteins have been documented. Three separate studies reported distinct chromosomal rearrangements whose breakpoints are located within SKA3 and DDX10 [85,86,87]. SKA3, on chromosome 13q11.12, encodes the spindle and kinetochore-associated complex-subunit 3, a 412 amino acid member of the SKA complex, which is essential for stable spindle attachment, proper chromosome segregation, and mitotic progression [87,88]. Jiao et al. [85] first identified SKA3::DDX10 fusion in two out of fifteen patients with hormone receptor–negative breast cancer. The DDX10 (NM_004398.4) breakpoint was located within intron 10, and the SKA3 (NM_145061.6) breakpoint occurred in intron 4 or in exon 4. More recently, whole-genome sequencing (WGS) and high-resolution SNP array analyses identified the rearrangement in two out of three cases with malignant pleural mesothelioma (MPM) [86]. Specifically, these were inter-chromosomal rearrangements in which a nearly complete sequence of SKA3 was inserted between introns 10 and 11 of the DDX10 gene, likely a retrotransposition process, although the exact mechanism remains unclear [86]. An additional SKA3::DDX10 fusion was detected in one case of primary pigmented papillary epithelial tumor of the sella (PPPET) [87] with breakpoints within exon 1 of SKA3 and intron 10 of DDX10. This fusion was out-of-frame, preventing functional protein production and likely causing partial or complete loss of DDX10 function.

In hematologic disorders, DDX10 contributes to disease through its fusion with *NUP98*, a gene located on chromosome 11p15.4. *NUP98* is a component of the nuclear pore complex known for being rearranged with more than 30 partner proteins [89,90]. The NUP98::DDX10 fusion was first identified in four cases of myeloid malignancies (cases 1–4 in Table 1) [91,92]. To date, 25 cases have been reported (Table 1), and here we contribute an additional, unpublished case (n. 24 in Table 1) [91,92,93,94,95,96,97,98,99,100,101,102,103,104,105,106]. Cases include 13 de novo (nos. 3, 4, 6, 7, 9, 10, 13, 15–18, 21, 25) and 7 therapy-related (nos. 1, 2, 5, 8, 11, 12, 22) myeloid neoplasms, including myelodysplastic syndrome (MDS, n = 5), acute myeloid leukemia (n = 15), chronic myeloid leukemia in accelerated phase (AP-CML, n = 1), chronic myelomonocytic leukemia (CMML = 1), acute leukemia of ambiguous lineage not otherwise specified (ALAL-NOS, n = 1) [91,92,93,94,95,96,97,98,99,100,101,102,103,104,105,106]. The clinical landscape of NUP98::DDX10 rearrangements reveals a distinct male predominance (male/female ratio of 18/7) and a broad age distribution, though the median age of 38 years at diagnosis (range 0.08–83 years, Table 1) highlights a significant impact on young and middle-aged adults. This demographic profile and the aggressive clinical course are highly characteristic of *NUP98* rearrangements, which are frequently associated with younger populations and represent a distinct subgroup of high-risk myeloid malignancies [89]. Furthermore, the significant incidence of therapy-related cases within this cohort (28%) reinforces the association between *NUP98* fusions and genomic instability following cytotoxic exposure.

In all cases, the rearrangements were detected using conventional and/or molecular cytogenetics techniques, including inv(11)(p15q22) in 21 out of 24 cases, ins(11)(p11q23) in 2 cases, and t(11;11)(p15;q22) in one case [104] (Table 1). RT-PCR confirmed the expression of the fusion transcript in all cases. Four in-frame isoforms of the NUP98::DDX10 transcript have been characterized based on the breakpoints. Type I fusion between exon 12 of *NUP98* (NM_139131.5) and exon 6 of DDX10 (NM_004398.4) was reported in 2 adult therapy-related AML patients [91,100]. Type II, between exon 14 of *NUP98* and exon 7 of DDX10, is the most common, accounting for 66.7% (16/24) of reported cases [91]. Type III, between exon 15 of *NUP98* and exon 7 of DDX10 [101], and type IV, between exon 14 of *NUP98* and exon 13 of DDX10 [104], were found in one case, respectively [101,104].

**Figure 3:**
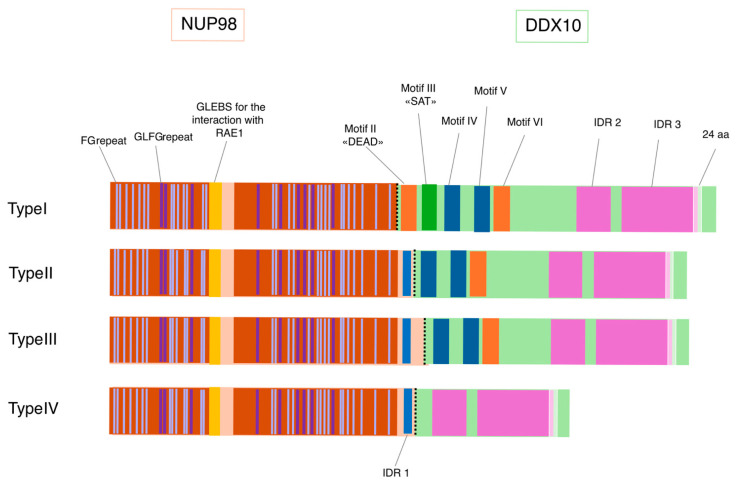
**Schematic representation of the four NUP98-DDX10 fusion protein types highlighting the 24aa.** All fusion proteins retain the N-terminal region of *NUP98*, which includes the two FG repeat regions (30 FG and 9 GLFG repeats), the GLEBS domain, and, for the fusion types II, III, and IV, also the IDR1. Additionally, the fusions contain the C-terminal part of DDX10, retaining different motifs/regions according to the fusion types: Type I—motif II-III-IV-V-VI and IDR2-3; Type II—motif IV-V-VI and IDR2-3; Type III—motif IV-V-VI and IDR2-3; Type IV—IDR2-3.

Regardless of the genomic breakpoints, NUP98::DDX10 fusion consistently retains the entire N-terminal region of *NUP98*, containing FG (Phe-Gly) and GLFG (Gly-Leu-Phe-Gly) repeats [107], fused to the C-terminal portion of DDX10 [107]. The latter, maintain specific motifs/regions, according to the four different isoforms (Figure 3): Type I fusion protein retains motif II-III-IV-V-VI and IDR2-3; Type II retains motif IV-V-VI and IDR2-3; Type III retains motif IV-V-VI and IDR2-3; Type IV retains IDR2-3.

This configuration is the arrangement generally considered leukemogenic for *NUP98* fusion oncoproteins. However, reciprocal DDX10::NUP98 transcripts were also detected in 6 out of 7 investigated cases with fusion types I, II and III [91,95,99,101]. The detection of reciprocal DDX10::NUP98 transcripts raises intriguing questions regarding their potential contribution to leukemogenesis. Particularly, in Type II and III isoforms, the conservation of key functional domains, such as the DEAD-box and the SAT domain, suggests that these reciprocal products might retain enzymatic activity and could exert a dominant-negative effect, competing with wild-type DDX10 for RNA substrates or nucleolar partners. Conversely, Type I transcripts lack these essential motifs, suggesting they may be catalytically impaired. Nevertheless, it is important to note that the actual translation and stability of these reciprocal fusion proteins have not yet been investigated or demonstrated in clinical samples, leaving their definitive biological role an open question. Single-cell transcriptomic data for NUP98::DDX10 cases are currently unavailable due to the rarity of this rearrangement. However, single-cell fluorescence in situ hybridization (FISH) analysis using Fluorescence Immunophenotype and Interphase Cytogenetics as a Tool for Investigation of Neoplasms (FICTION) performed showed that the translocation involves the CD34^+^/CD133^+^ hematopoietic precursors, suggesting that leukemogenic transformation likely originates at an early progenitor stage, as observed in other *NUP98* fusion–driven leukemias [108].

**Table 1:** Description on NUP98::DDX10 cases in literature and an additional unpublished case.

No.	Sex/Age	Disease Type	Chromosomal Karyotype	Fusion Gene Type/Reciprocal Fusion Transcript	Coexisting Gene Mutations	Treatment/Outcome	Reference
**1**	M/61	AML-M4	46, XY, t(3;5)(p13;q35),inv(11)(p15q22)[9]/46,XY[1]	I/Yes	NA	NA/NA ^1^	[91,92]
**2**	F/4	MDS (RA)	46,XX,inv(11)(p15q22.3)[16]	II/Yes	NA	SCT/CR ^2^	[91,92,93]
**3**	M/10	AML-M1	47,XY,inv(11)(p15q22),+21[16]	II/NF	NA	NA/NA	[91,92]
**4**	M/7	MDS (RAEBT)	46,XY,inv(11)(p15q22)[20]	II/Yes	NA	NA/NA	[91,92]
**5**	M/51	CMML	46,XY,inv(11)(p15q22)	II/Yes	NA	NA/Progressed to AML-M4—D ^3^	[94,95]
**6**	M/58	AP-CML	46,XY,t(9;22)(q34;q11),inv(11)(p15q22)[15]	II/NA	NA	TKI + Chemotherapy/Progressed to a blast crisis—D	[96]
**7**	M/6	AML	46,XY,inv(11)(p15q21)[3]/46,XY,idem,der(17)t(17;?)(q?11;?)[10]/46,XY,idem,del(2)(p?22),der(17)t(17;?)(p?11;?)[5]/46,XY[2]	II/NA	NA	Chemotherapy/D	[97]
**8**	M/18	AML-M4	46,XY, inv(11)(p15q22)[20]	II/NA	NA	Chemotherapy/NA	[97]
**9**	M/0.08	AML-M5	46,XY,ins(11)(p11q23)[14]/46,XY[2]	II/NA	NA	NA/NA	[98]
**10**	F/60	AML-M4	46,XX,inv(11)(p11q23),del(12)(p13)[20]	NA/NA	NA	NA/NA	[98]
**11**	F/47	AML	46,XX,inv(11)(p15q22)[17]/46,XX[6]	NA/NA	NA	NA/NA	[98]
**12**	F/55	MDS	46,XX,t(11;11)(p15;q22)[9]/46,XX[11]	II/NA	NA	NA/NA	[98]
**13**	M/12	AML-M5b	46,XY,inv(11)(p15q22)	II/Yes	NA	SCT/CR	[99]
**14**	M/32	AML-M6	47,XY,+8,inv(11)(p15q22)	I/NA	*N-RAS*	Chemotherapy/D	[100]
**15**	M/39	AML-M4	30~45,XY,-1[3],-9[4],10[4],inv(11)(p15q22)[10],-15[3],-17[4],-18[4],-19[4],-20[4],-21[4][cp10]	II; III/Yes	NA	Chemotherapy/D	[101]
**16**	M/4	AML-M5	46,XY,inv(11)(p15q22)[19]/46,XY[1]	II/NA	NF ^4^	Chemotherapy/CR	[102,106]
**17**	F/31	AML-M5b	46,XX,inv(11)(p15q22)[19]	II/NA	*KRAS*, *NRAS*	Chemotherapy/CR	[104]
**18**	M/38	ALAL-NOS	der(2)t(2;11)(p11.2;p11.2)?inv(11)(p15q23),der(11)t(2;11)?inv(11),del(12)(p12), del(13)(q12q14), and del(14)(q24)	IV/NA	*CREBBP*, *NF1*, *CTCF*, *PHF6*	SCT/CR	[104]
**19**	M/1.3	NA	der(11)ins(11;11)(p15; q21q23)	II/NA	NA	NA/R ^5^-D	[105]
**20**	M/11.8	NA	inv(11)(p15q22)	NA/NA	NA	SCT/R	[105]
**21**	M/45	AML-M5	46,XY,inv(11)(p15q22)[12]/46,idem,del(5)(q22q33)[5]/46,XY[3]	II/NA	*KRAS*, *WT1*	Allogenic SCT	[106]
**22**	F/50	MDS	46,XX,inv(11)(p15q22)[14]/46,XX[6]	II/NA	NA	NA/D	[106]
**23**	F/-	AML	47, XX, add(6)(p21),+8, inv(11)(p15q22)[19]/46, XX[1]	NA/NA	NA	NA/NA	[103]
**24**	M/83	AML	46,XY,inv(11)(p15q22)[12]/46,XY[8]	NA/NA	*BCOR*, *WT1*, *PTPN11*, *NRAS*	NA/NA	Unpublished
**25**	M/80	MDS	46,XY,t(3;3)(q21;q26)[7/37]/46,XY,t(3;3)(q21;q26),inv(11)(p15q22-q23)[4/37]/46,XY[26/37]	NA/NA	NF	NA/NA	[109]

^1^ NA: Not Applicable; ^2^ CR: Complete Remission; ^3^ D: Dead; ^4^ NF: Not Found; ^5^ R: Remission.

Evidence suggests that the NUP98::DDX10 fusion may act by disrupting the synthesis of proteins involved in myeloid lineage differentiation, through aberrant nucleocytoplasmic mRNA transport and altered ribosome assembly [91]. The transforming potential and transcriptional regulation properties of DDX10 in the fusion have been attributed to motif VI (YIHRAGRTAR) and to a 24 amino acid stretch located within the protein C-terminal [42,110]. Introducing mutations in the three conserved arginine residues within motif VI (NUP98::DDX10/3Q) was found to slow the proliferation of human hematopoietic precursor cells (hCD34^+^) in long-term cultures, to promote myeloid differentiation in colony-forming cell (CFC) assays, and to reduce the ability of the fusion to activate target promoters [42].

Recent studies have shown that LLPS contributes to *NUP98* fusion–driven leukemogenesis by promoting the formation of nuclear condensates that alter transcriptional programs and facilitate leukemic transformation [111]. The ability of NUP98::DDX10 to undergo LLPS likely stems from IDRs present in both *NUP98* [111] and DDX10 [43] (Figure 3). However, Shima et al. [110] demonstrated that LLPS alone is insufficient to drive leukemic transformation. They identified a 24–amino acid sequence (SNSEVEDVGPTSHNRKKARWDTLE) within the IDR3 of DDX10 that is specifically required for cell immortalization and leukemogenesis but dispensable for membraneless condensate formation. Notably, these 24 amino acids are conserved across all four isoforms, suggesting that they may represent a shared pathogenic element contributing to leukemia development independent of the specific fusion isoform (Figure 3). This sequence mediates interaction with the nucleolar protein NOL10, and their association within condensates is essential for stabilizing ATF4 (Activating Transcription Factor 4) mRNA. This stabilization activates the serine biosynthesis pathway, a metabolic process dysregulated in NUP98::DDX10 leukemia and other cancers [110,112].

Mutations on DDX10, with potential prognostic significance, have also been identified in MDS [113]. Waheed et al. [113] examined 47 treatment-naïve primary MDS patients to evaluate the prognostic significance of gene mutations on overall survival. They identified two DDX10 variants, a non-frameshift deletion (p.Asp788del) and a missense mutation (p.Arg509Cys), in four patients. DDX10 emerged as one of the most frequently mutated genes in the cohort and was associated with low-risk and favorable prognosis, although its prognostic value was not consistently supported across other studies [114,115,116,117].

## 5. DDX10 as a Diagnostic Biomarker and Target for Therapy

### 5.1. DDX10 as a Tumor-Selective Biomarker in Extracellular Vesicles (EVs)

The growing body of evidence supporting the role of DDX10 in oncogenesis has prompted interest in its clinical exploitation. This section explores emerging strategies that leverage DDX10 for precision oncology, ranging from non-invasive diagnostics to the development of targeted therapies.

The detection of tumor-derived biomarkers in extracellular vesicles (EVs), including exosomes, represents a rapidly evolving strategy for non-invasive cancer diagnostics [118]. In this context, DDX10 has emerged as a promising candidate, given its selective presence on the surface of EVs derived from breast cancer cell lines (MDA-MB-231 and MCF7) [119]. This cancer-specific enrichment suggests that DDX10 could serve as a tumor-selective EV marker, providing a non-invasive means to distinguish malignant from normal tissue states.

Importantly, DDX10 was not only identified within the EV cargo but was enriched on the vesicle surface, making it more amenable to capture and detection in circulating biofluids using antibody-based or affinity-based strategies [120]. This feature enhances its utility as a liquid biopsy marker [121], potentially enabling early detection, real-time disease monitoring, and even prediction of therapy response [122,123]. As previously mentioned, in OSCC DDX10 directly facilitates the packaging of PD-L1 into exosomes, suggesting that its detection in biofluids might also serve as a surrogate indicator of tumor immunosuppressive activity [78].

### 5.2. Pharmacological Targeting of DDX10: A New Frontier in Cancer Therapy

While DDX10 has recently emerged as a potential therapeutic vulnerability, pharmacological targeting of this RNA helicase remains at a very early stage. To date, compound 5o represents the only reported small-molecule antagonist of DDX10, identified through a phenotypic screening of selenium-based heterocycles originally developed for anticancer activity rather than through a target-driven drug discovery strategy [60]. 5o emerged as a potent anticancer molecule in NCI-H460 lung cancer cells, which significantly reduced migration and invasion by blocking EMT process, as evidenced by decreased N-cadherin and vimentin levels and increased E-cadherin expression [84,124,125,126,127].

**Figure 4:**
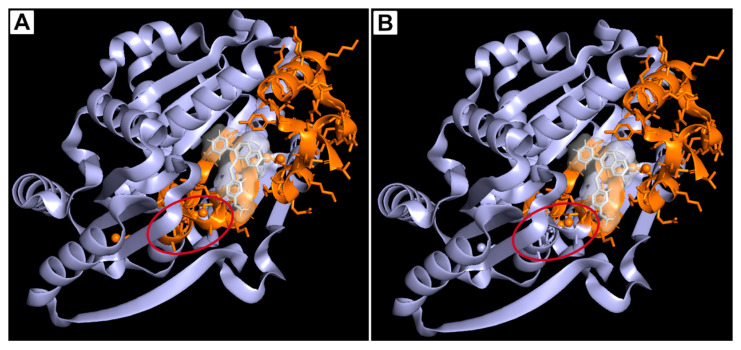
**Binding interactions of compound 5o within the ADP binding pocket of DDX10.** (**A**) Compound 5o, colored in white, positioned in the ADP binding pocket with all four conserved motifs (Q, I, II, and VI colored in orange) highlighted; a red circle emphasizes motif VI that does not participate in the binding, as the natural site for ADP. (**B**) Compound 5o in the ADP binding pocket with only motifs Q, I, and II highlighted in orange; motif VI is not highlighted (same color as the protein), and a red circle indicates its lack of interaction, represented by a lack of orange coloring. Avogadro software (https://avogadro.cc/, accessed on 25 June 2025) was used to generate a 5o 3D visualization based on the chemical structure reported by Xu et al. [60]. The obtained structure was imported into PyMOL and modeled with DDX10.

Moreover, 5o induced DNA damage, indicated by enhanced phosphorylation of γ-H2A.X [128,129] and apoptosis [130], with increased levels of the pro-apoptotic protein BAX and cleaved caspase-3, coupled with downregulation of the anti-apoptotic marker Bcl-2. The therapeutic relevance of targeting DDX10 was further supported by siRNA-mediated knockdown, which recapitulated the effects of compound 5o on cell viability, and by xenograft models confirmed the anticancer efficacy of 5o. Molecular docking revealed that 5o forms hydrogen bonds with Gly-118 and Lys-119 within DDX10’s ADP-binding pocket, implying a competitive inhibition mechanism (Figure 4). Competitive binding assays also demonstrated that 5o disrupts ADP-DDX10 interactions, reinforcing its role as a potent DDX10 antagonist [60].

While these findings support direct target engagement, the absence of comprehensive selectivity profiling leaves open the possibility of off-target effects, particularly given the high structural conservation among DEAD-box RNA helicases. In addition, the physicochemical and pharmacokinetic properties of 5o have not been systematically optimized, and potential liabilities associated with its selenium-containing scaffold remain unexplored.

## 6. DDX10 in Perspective: Challenges and Opportunities

DDX10 has emerged as a multifunctional DEAD-box RNA helicase with pivotal roles in ribosome biogenesis, RNA metabolism, and antiviral defense, extending beyond its canonical enzymatic activities. Its dysregulation in both solid and hematological malignancies underscores its involvement in oncogenic transformation and tumor progression, supporting its relevance as a potential biomarker and therapeutic target. In this context, the fortuitous identification of compound 5o provides a proof-of-principle for the druggability of DDX10, while also highlighting the early and largely exploratory nature of pharmacological efforts in this area.

Despite recent advances, DDX10 remains underexplored compared to other RNA helicases, leaving a significant knowledge gap, particularly regarding the ‘DDX10 nexus’ between phase separation and metabolic control. Addressing this gap is essential for the rational development of selective inhibitors and the identification of predictive biomarkers. By exploring these context-specific functions, this review aims to provide the foundation necessary to enable future translational applications.

## Data Availability

No new data have been created for this review.

## Abbreviations

The following abbreviations are used in this manuscript:

DDX	DEAD-box
LLPS	Liquid–liquid phase separation
ATP	Adenosine Triphosphate Protein
SF2	Helicase Super Family 2
DEAD	Asp-Glu-Ala-Asp
RecA	Recombinase-A
IDRs	Intrinsically Disordered Regions
DFC	Dense Fibrillar Component
GC	Granular Component
mESCs	mouse Embrionic Stem Cells
PRRSV	Porcine Reproductive and Respiratory Syndrome Virus
PCV3	Porcine Circo Virus type 3
ISGs	Interferon-Stimulated Genes
NLS	Nuclear Localization Signal
HIV-1	Human Immunodeficiency Virus
OSCC	Oral Squamous Cell Carcinoma
CRC	Colo-Rectal Cancer
LUAD	Lung Adeno carcinoma
PDAC	Pancreatic Ductal Adeno carcinoma
DLBCL	Diffuse Large B-Cell Lymphoma
ATG	Autophagy-related Gene
FBL	Fibrillarin
EMT	Epithelial–Mesenchymal Transition
ICIs	Immuno-Checkpoint Inhibitors
SKA3	Spindle and Kinetochore-Associated complex-subunit 3
WGS	Whole-Genome Sequence
MPM	Malignant Pleural Mesothelioma
PPPET	Primary Pigmented Papillary Epithelial Tumor of the Sella
FG	Phe-Gly
GLFG	Gly-Leu-Phe-Gly
FISH	Fluorescence In Situ Hybridization
FICTION	Fluorescence Immunophenotype and interphase Cytogenetics as a Tool for Investigation Of Neoplasms
NHL	Non-Hodgkin Lymphoma
CHOP	cyclophosphamide, doxorubicin, vincristin, prednisone
Mit	Mitoxantrone
Etop	Etoposide
Pep	Pepreomycin
Pred	Prednisone
RT	Radiotherapy
AML	Acute Myeloid Leukemia
NA	Not Available
ALL	Acute Lymphoblastic Leukemia
Vcr	Vincristine
Dox	Doxorubicin
Dnr	Daunorubicin
L-Asp	I-asparaginase
MDS	Myelodysplastic Syndrome
RA	Refractory Anemia
RAEBT	Refractory Anemia with Excess Blasts in Transformation
I-TOPOII	Topoisomerase II Inhibitors
EGCT	Extratesticular Germ Cell Tumor
CMML	Chronic MyeloMonocytic Leukemia
AP-CML	Accelerated Phase of Chronic Myeloid Leukemia
TKI	Tyrosine Kinase Inhibitor
APL	Acute Promyelocytic Leukemia
BC	Breast Cancer
AL-NOS	Acute leukemia, Not Otherwise Specified
SCT	Stem Cell Transplantation
OC	Ovarian Cancer
CFC	Colony Forming Cell
ATF4	Activating Transcription Factor 4
GLEBS	Gle 2-Binding Sequence
EVs	Extracellular Vesicles
